# Graft function and pregnancy outcomes after kidney transplantation

**DOI:** 10.1186/s12882-022-02665-2

**Published:** 2022-01-12

**Authors:** Anke Schwarz, Roland Schmitt, Gunilla Einecke, Frieder Keller, Ulrike Bode, Hermann Haller, Hans Heinrich Guenter

**Affiliations:** 1 Department of Nephrology, Hannover Medical School, Carl-Neuberg Strasse 1, D-30625 Hannover, Germany; 2 Division of Nephrology, Ulm University, Ulm, Germany; 3 Department of Visceral Surgery and Transplantation, Hannover Medical School, Hannover, Germany; 4 Department of Gynecology and Obstetrics, Hannover Medical School, Hannover, Germany

**Keywords:** Pregnancy, Kidney transplantation, Graft function, Graft survival, Preeclampsia

## Abstract

**Background:**

After kidney transplantation, pregnancy and graft function may have a reciprocal interaction. We evaluated the influence of graft function on the course of pregnancy and vice versa.

**Methods:**

We performed a retrospective observational study of 92 pregnancies beyond the first trimester in 67 women after renal transplantation from 1972 to 2019. Pre-pregnancy eGFR was correlated with outcome parameters; graft function was evaluated by Kaplan Meier analysis. The course of graft function in 28 women who became pregnant after kidney transplantation with an eGFR of < 50 mL/min/1.73m^2^ was compared to a control group of 79 non-pregnant women after kidney transplantation during a comparable time period and with a matched basal graft function.

**Results:**

Live births were 90.5% (fetal death *n* = 9). Maternal complications of pregnancy were preeclampsia 24% (graft loss 1, fetal death 3), graft rejection 5.4% (graft loss 1), hemolytic uremic syndrome 2% (graft loss 1, fetal death 1), maternal hemorrhage 2% (fetal death 1), urinary obstruction 10%, and cesarian section. (76%). Fetal complications were low gestational age (34.44 ± 5.02 weeks) and low birth weight (2322.26 ± 781.98 g). Mean pre-pregnancy eGFR was 59.39 ± 17.62 mL/min/1.73m^2^ (15% of cases < 40 mL/min/1.73m^2^). Pre-pregnancy eGFR correlated with gestation week at delivery (*R* = 0.393, *p* = 0.01) and with percent eGFR decline during pregnancy (*R* = 0.243, *p* = 0.04). Pregnancy-related eGFR decline was inversely correlated with the time from end of pregnancy to chronic graft failure or maternal death (*R* = -0.47, *p* = 0.001). Kaplan Meier curves comparing women with pre-pregnancy eGFR of ≥ 50 to < 50 mL/min showed a significantly longer post-pregnancy graft survival in the higher eGFR group (*p* = 0.04). Women after kidney transplantation who became pregnant with a low eGFR of > 25 to < 50 mL/min/1.73m^2^ had a marked decline of renal function compared to a matched non-pregnant control group (eGFR decline in percent of basal eGFR 19.34 ± 22.10%, *n* = 28, versus 2.61 ± 10.95%, *n* = 79, *p* < 0.0001).

**Conclusions:**

After renal transplantation, pre-pregnancy graft function has a key role for pregnancy outcomes and graft function. In women with a low pre-pregnancy eGFR, pregnancy per se has a deleterious influence on graft function.

**Trial registration:**

Since this was a retrospective observational case series and written consent of the patients was obtained for publication, according to our ethics’ board the analysis was exempt from IRB approval. Clinical Trial Registration was not done. The study protocol was approved by the Ethics Committee of Hannover Medical School, Chairman Prof. Dr. H. D. Troeger, Hannover, December 12, 2015 (IRB No. 2995–2015).

## Background

The first pregnancy after kidney transplantation occurred in 1956. Edith Helm had received a kidney from her identical twin sister, when both were 21 years old [1]. Actually, this case was the world’s third kidney transplant and Edith Helm became pregnant after the third post-transplant menstrual cycle [2]. She had a second pregnancy later and lived to age 76 years without immunosuppression. Pregnancy after non-living donor transplantation under immunosuppression was reported 11 years later [3]. Forty years thereafter, pregnancies after solid-organ transplants still are high-risk, but now have become one of the expected benefits of transplantation [4].

In Hannover, the first pregnancy extending beyond the first trimester after non-living kidney transplantation occurred in 1972, but resulted in stillbirth. Thus, the first pregnancy with a living child occurred in 1977. We report on all beyond first trimester pregnancies at our institution since 1972. In addition to general outcomes for mother and child, our main interest was the function of maternal kidney graft resulting after pregnancy. In planned studies comparing graft function of transplanted women with and without pregnancy, usually no difference was found since renal function of the women was in most cases excellent [5]. However, our purpose was to analyze the outcomes of all women including those with estimated glomerular filtration rate (eGFR) < 50 mL/min/1.73m^2^. This viewpoint represents “real life” and may assist in counseling women after kidney transplantation. In order to specify our question regarding harm to graft function by pregnancy per se in patients with suboptimal pre-pregnancy graft function, we compared women after kidney transplantation with pregnancy and low renal function to a control group of transplanted women who did not become pregnant but were matched for similar graft function.

## Methods

All women after kidney transplantation with a pregnancy beyond the first trimester were included in this retrospective observational study; these women had been transplanted at Hannover Medical School since 1970. Data were obtained from the medical records. The study protocol was approved by the Ethics Committee of Hannover Medical School, Chairman Prof. Dr. H. D. Troeger, Hannover, December 12, 2015 (IRB No. 2995–2015). Written informed consent of the patients to participate in the study was obtained. Principles as outlined in the “Declaration of Helsinki” and the “Declaration of Istanbul on Organ Trafficking and Transplant Tourism” were respected.

We inspected details of the clinical course of the women, as there are basal kidney disease, number of transplantations, duration of renal insufficiency before transplantation, renal function, hypertension and proteinuria prior and during pregnancy, immunosuppression, and outcomes of the mothers and their grafts after pregnancy. We considered details of pregnancy such as duration of gestation, kind of delivery, complications during pregnancy and after delivery. We recorded offspring outcomes during pregnancy and after delivery. Graft function and offspring outcomes from the period January 1^st^, 1972 up to December 31^st^, 1999 were compared to that of January 1^st^, 2000 up to December 31^st^, 2019 to control for time-related era effect. Some of the patients reported here appeared earlier in a report comparing pregnancy outcomes in renal and hepatic transplantation [6].

Glomerular filtration rate (eGFR ml/min/1.73m^2^) was estimated by the CKD-EPI creatinine equation (Chronic Kidney Disease Epidemiology Collaboration) [7] before pregnancy and 3–4 months after delivery. Correlations were calculated (MS Excel) between pre-pregnancy eGFR and weeks of gestation until delivery; between pre-pregnancy eGFR and percent of eGFR decline after pregnancy; and between percent of eGFR decline after pregnancy and time interval from delivery to graft failure after pregnancy in months (including death of the mother with a functioning graft). Furthermore, we inspected correlations between age of the transplant, including age of the donor at donation, and percent loss of eGFR by pregnancy.

Kaplan–Meier curves were plotted separately for women with a pre-pregnancy eGFR of ≥ 50 mL/min/1.73m^2^ and those with eGFR < 50 ml/min/1.73m^2^. Patient death with a functioning graft was classified as graft failure. Patients lost to follow up with a functioning graft were censored at time of last visit. Kaplan–Meier analysis was carried out using GraphPad Prism (GraphPad software). Mantel Cox log rank test was used for comparison of graft survival between groups. Student’s t-test, Mann–Whitney U-test, and chi-square test were used for comparisons between groups, as appropriate. Differences with *p* < 0.05 were considered statistically significant.

For the group of women with pregnancy after kidney transplantation and a low pre-pregnancy eGFR of > 25 to < 50 mL/min/1.73m^2^ (study group, basal eGFR, *n* = 28), we established a control group of women after kidney transplantation who did not become pregnant but had a similarly low eGFR. The control group consisted of all adult patients with kidney transplantation in Hannover between January 1, 2000 to December 3, 2017 (*n* = 2819). We selected women aged 18 to 40 years with the potential of childbearing who did not become pregnant after transplantation (*n* = 299). We further selected women who were transplanted at least 5 years with eGFR between > 25 to < 50 mL/min/1.73m^2^ at post-transplant year 4 (control group, basal eGFR, *n* = 79). The change in pre-pregnancy graft function of the study group (basal eGFR) to post-pregnancy graft function 3 to 4 months after pregnancy (one-year eGFR) was compared to the change of post-transplant graft function in the non-pregnant control group at the end of year 4 (basal eGFR) to post-transplant graft function at the end of year 5 (one-year eGFR). The change in the one-year eGFR was expressed as percent of the basal eGFR. Since the mean transplantation-to-conception time of the pregnancy group was 6.5 ± 5.33 years (Table 1), we elected to compare the decrease in graft function in the pregnancy group to year 4 eGFR to one-year 5 time period of the control group.

**Table 1:** Demographic data of 67 mothers, 92 pregnancies and 95 offsprings

**Course of kidney disease and pregnancy of the mothers:**	
Duration of dialysis (yrs)	2.85 ± 2.37 (0–9)
Preemptive transplantation (%)	*n* = 4 (5.97)
Living-transplant (%)	*n* = 23 (34.33)
Transplantation before the age of 18 years (%)	*n* = 14 (20.9)
Age of the mothers at transplantation (yrs)	23.72 ± 7.18 (6.5–35)
Post-transplant hypertension pre-pregnancy (%)	*n* = 52/71 (73)
Age of the mothers at conception (yrs)	29.6 ± 4.7 (15–39)
Time from transplantation to conception (yrs)	6.51 ± 5.33 (0.5–24.75)
Transplant age at conception including donor age (yrs)	39.75 ± 14.6 (9.5–67.2)
**Number of transplants:**	
Pregnancy with the 1^st^ transplant (%)	*n* = 82 (89.13) 85 deliveries (twins 3)
Pregnancy with the 2^nd^ transplant (%)	*n* = 10 (10.87) 10 deliveries
re-Tx once after that with pregnancy (%)	*n* = 11 (11.96)
re-Tx twice after that with pregnancy (%)	*n* = 4 (4.35)
**Additional extrarenal transplants:**	
Combined pancreas-kidney-transplantation	*n* = 3^a^
Liver before kidney transplantation	*n* = 2
**Timing of pregnancy after transplantation:**	
Pregnancy in yr 1–5 after transplantation (%)	*n* = 44 (47.83)
Pregnancy in yr 6–10 after transplantation (%)	*n* = 28 (30.43)
Pregnancy in yr 11–20 after transplantation (%)	*n* = 17 (18.48)
Pregnancy beyond yr 20 after transplantation (%)	*n* = 3 (3.26)
**Number of pregnancies and deliveries per woman beyond the first trimester:**	
1 Pregnancy (%)	*n* = 46 (50) 48 deliveries (twins 2)
2 Pregnancies (%)	*n* = 18 (39.13) 37 deliveries (twins 1)
3 Pregnancies (%)	*n* = 2 (6.52) 6 deliveries
4 Pregnancies (%)	*n* = 1 (4.35) 4 deliveries
**Immunosuppression during pregnancy**	
Azathioprin, prednisolone (%)	*n* = 11 (11.96)
Cyclosporine, prednisolone (%)	*n* = 40 (43.48)
Cyclosporine, azathioprine, prednisolone (%)	*n* = 12 (13.04)
Tacrolimus, prednisolone (%)	*n* = 11 (11.96)
Tacrolimus, azathioprine, prednisolone (%)	*n* = 18 (19.57)
**Mean pre-pregnancy eGFR:**	
All pregnancies	59.39 ± 17.62 mL/min (*n* = 88)
Pre-pregnancy eGFR ≥ 60 mL/min/1.73m^2^	73.91 ± 10.33 (*n* = 44)
Pre-pregnancy eGFR ≥ 50-60 mL/min/1.73m^2^	54.16 ± 3.95 (*n* = 16)
Pre-pregnancy eGFR ≥ 40-50 mL/min/1.73m^2^	45.79 ± 3.36 (*n* = 14)
Pre-pregnancy eGFR < 40 mL/min/1.73m^2^	33.33 ± 4.29 (*n* = 14)
**Fetal outcome:**	
gestational age at birth (weeks)	34.44 ± 5.02 weeks (*n* = 88)
live-birthrate	*n* = 86 (90.53%)
< 37 SSW	*n* = 45 (von 76)
< 34 SSW	*n* = 20 (von 76)
< 28 SSW	*n* = 2 (von 76)
Cesarian section (%)	*n* = 67 von 88 (76.1)
Mean birth weight	2146.75 ± 926.14 (*n* = 82)
Mean birth weight (only live birth)	2322.26 ± 781.98 (*n* = 79)
≤ 2500	*n* = 40 (of 79)
≤ 1500	*n* = 21 (of 79)
≤ 1000	*n* = 6 (of 79)

^a^one pancreas failed before pregnancy

*yrs* years, *fct* function

## Results

In Hannover, 67 women after kidney transplantation had 92 pregnancies beyond the first trimester and 95 deliveries since 1972. The number of pregnancies over the decades was rather stable after 1981 (2–3 pregnancies beyond the first trimester per year). Twenty-one of 67 women (31.34%) had more than one pregnancy. Sixty women had 82 pregnancies and 85 deliveries during the time of their first kidney transplantation (3 pairs of twins), while 7 women had 10 pregnancies and deliveries with their second kidney transplant. Before 1972 no pregnancy beyond the first trimester existed; pregnancies usually were terminated by induced abortion as preventing measure of damage to the graft. Fifteen women had to be re-transplanted once and 4 twice because of graft failure during the years following pregnancy. Fifty-four of 67 women (80.6%) were older than 30 years getting pregnant; and in 70 of 92 pregnancies (76.1%), pregnancy occurred more than 36 months after transplantation. In 45 of 92 pregnancies (48.9%), the age of the kidney transplant was more than 40 years old (duration since transplantation plus age of the donor at donation), and in 7 of 92 pregnancies (7.6%), transplant age at conception and delivery was even more than 60 years.

We have documented that since 2000, 66 women after counselling for pregnancy had their medications altered considering the intention to become pregnant. Forty women (60.6%) since that time experienced 48 pregnancies extending beyond the first trimester (including 3 fetal deaths). However, the other 26 women (39.4%) during the same period of time (20 years) were unable to conduct a pregnancy beyond the first trimester. The number of miscarriages during the first trimester could not be assessed here, since this parameter was not consistently reported to the transplant center by the women or their obstetricians.

Basal kidney disease of the women with pregnancy was nephronophthisis *n* = 2, Alport’s syndrome 2, medullary cystic disease 1, primary hyperoxaluria 1, cystinosis 1, congenital kidney and urinary tract abnormality 13, primary amyloidosis 1, diabetes 4, malignant nephrosclerosis 4, pyelonephritis 3, IgA nephropathy 9, membranoproliferative glomerulonephritis 4, focal segmental glomerulosclerosis 5, membranous glomerulonephritis 1, polyarteritis nodosa 1, ANCA vasculitis 1, lupus nephritis 2, and diagnosis not clarified 12. Demographic data of the women, their details of transplantation and time of dialysis before transplantation, their pregnancies and deliveries, as well as data of fetal outcomes are given in Table 1 and 2. One mother with congenital anomaly of kidney and urinary tract (CAKUT) inherited the condition to her son, who subsequently developed end-stage renal disease as a young adult and required renal transplantation as well.

**Table 2:** Complications of pregnancy; graft loss during and up to 1 year after pregnancy 4 (4.4%); fetal death during and up to 1 month after delivery 9 (9.5%)

Pre-eclampsia-related problems	*n* = 22 (23.91%) acute graft loss 1 fetal death 3 (3/22 with vs 6/73 without preeclampsia *p* = 0.02)
placenta previa	*n* = 2 (2.17%) fetal death 1
hemolytic-uremic syndrome of the mother	*n* = 2 (2.17%; DD severe HELLP syndrome) acute graft loss 2 (1 irreversible, 1 partially reversible after 7 mos of dialysis) fetal death 1
spontaneous maternal retroperitoneal bleeding during pregnancy	*n* = 1^a^ fetal death 1
spontaneous maternal intraperitoneal bleeding during pregnancy	*n* = 1^b ^
transplant bleeding during cesarean section	*n* = 1
Not clarified intrauterine fetal death	*n* = 1
urinary obturation	*n* = 9 (9.78%)
intrahepatic cholestasis	*n* = 5 (5.43%)
rejections up to 1 year after pregnancy	*n* = 5 (5.43%)^c^ acute irreversible graft loss 1 (non-adherence of immunosuppression)
Terminating of pregnancy after 14 weeks because of maternal medical reasons^d^	*n* = 1^d^ fetal death 2 (twins)
mean pre-pregnancy eGFR:	loss of eGFR by pregnancy:
59.39 ± 17.62 mL/min/1.73 (all *n* = 88)	13.89 ± 20.41% of pre-pregnancy eGFR
> 60 mL/min/1.73m^2^ (*n* = 44)	11.63 ± 21.17%
≥ 50-60 mL/min/1.73m^2^ (*n* = 16)	11.12 ± 12.29%
≥ 40-50 mL/min/1.73m^2^ (*n* = 14)	16.41 ± 21.06%
< 40 mL/min/1.73m^2^ (*n* = 14)	22.76 ± 22.76%

^a^anticoagulation because of nephrotic-range proteinuria

^b^unknown reason; marked pre-existing abdominal scarred adhesions

^c^3 borderline rejections, 1 mild humoral rejection, 1 severe combined antibody-T-cell mediated rejection with graft loss by incompliance

^d^unintended pregnancy of twins noticed 3 months after transplantation, therefore no change of medication before pregnancy; additionally, suspected renal cell carcinoma of one native kidney

*Mos* months, *HELLP* hemolysis, elevated liver enzymes, and low platelets

Complications during pregnancy and up to one year after delivery resulted in graft loss in 4 cases (4.4%): suspected hemolytic uremic syndrome (HUS) in two women, severe preeclampsia in one case, and severe rejection by incompliance of taking immunosuppression in another case (Table 2). Rejections in general were seen in 5 cases (5.43%). Graft loss combined with fetal death was encountered in one woman of the two who had developed HUS. Genetic complement aberrations were not identified in the two women with HUS. Their underlying renal disease was biopsy-proven membrano-proliferative glomerulonephritis and focal segmental glomerulosclerosis, respectively. We have only limited data on urinary tract infections, since this rather frequent complication was mainly managed by the attending nephrologist outside the transplant center.

In 71 of 92 pregnancies, we had information about blood pressure preceding pregnancy (77.2%). Fifty-two patients (73.2%) had hypertension before 71 pregnancies and took 1.54 ± 0.78 antihypertensive drugs per day. In 42 women (62.7%) with 47 pregnancies (51.1%), we had information about proteinuria and hypertension during the last trimester. In 15 of these 47 pregnancies (31.9%) the women had proteinuria of ≥ 0.5 g/24 h; in 4 women ≥ 1 g/24 h (8.5%), and one woman had nephrotic range proteinuria. Thirty-six women with 47 pregnancies (76.6%) developed or aggravated hypertension during the third trimester and required at least one antihypertensive drug; 10 of them (21.3%) needed 2 drugs; 4 needed 3 (8.5%). Proteinuria and/or hypertension were not invariably related to symptomatic pre-eclampsia. In some cases, these features were difficult to differentiate from the underlying primary disease. Preeclampsia-like clinical symptoms were observed in 22 cases (23.9%, Table 2). Fetal or peripartum loss was observed in 3 out of 22 women with preeclampsia (13.64%) and only in 6 out of 73 women without (8.22%; *p* = 0.02). Preeclampsia was followed by acute irreversible graft failure in one case, while the child survived. The percent loss of eGFR by pregnancy compared to the pre-pregnancy value in general was not different between women with preeclampsia and those without (14.02 ± 18.68% vs 13.97 ± 21%; *p* = 0.50).

Complications resulting in fetal death were seen in 9 cases (9.5%; preeclampsia *n* = 3, placenta previa 1, hemolytic uremic syndrome 1, severe retroperitoneal hemorrhage under anticoagulation because of nephrotic-range proteinuria 1, and stillbirth for unclear reasons 1). Additionally, termination of pregnancy with twins had to be done because of a suspected maternal renal malignancy (*n* = 2) (Table 2). Live-birthrate was in these 92 pregnancies with 95 deliveries 90.5%. In 4 of 7 instances of fetal loss, pre-pregnancy eGFR in the women had been less than 40 mL/min/1.73m^2^, while fetal loss occurred in only 3 cases with eGFR of > 50 mL/min (*n* = 4 out of 14, 28.57%; versus 3 out of 57, 5.26%, *p* = 0.042). We excluded the elective abortion of a twin pair from this calculation. In the group with the lowest pre-pregnancy eGFR of < 40 mL/min/1.73m^2^, not only fetal death rate but also loss of eGFR after pregnancy was greatest (Table 2). Other parameters of maternal and fetal outcomes are given in Table 1 and 2.

Pre-pregnancy eGFR of women with pregnancy between January 1, 1972 up to December 31, 1999 (early period) was not different to that of women with pregnancy between January 1, 2000 to December 31, 2019 (late period) (57.73 ± 14.38, *n* = 37, versus 60.71 ± 20.16, *n* = 49; *p* = 0.764). The same was true for the change in pre-pregnancy to post-pregnancy eGFR (loss of eGFR 3–4 months after pregnancy in percent of pre-pregnancy eGFR: early period 15.94 ± 25.24%, *n* = 37, versus late period 12.22 ± 16.18%, *n* = 49; *p* = 0.408), and for fetal and perinatal death (6 out of 37, 16.22%, versus 3 out of 49, 6.12%; Chi-square 2.29; *p* = 0.13). Thus, we found no time-related era effect.

**Fig. 1:**
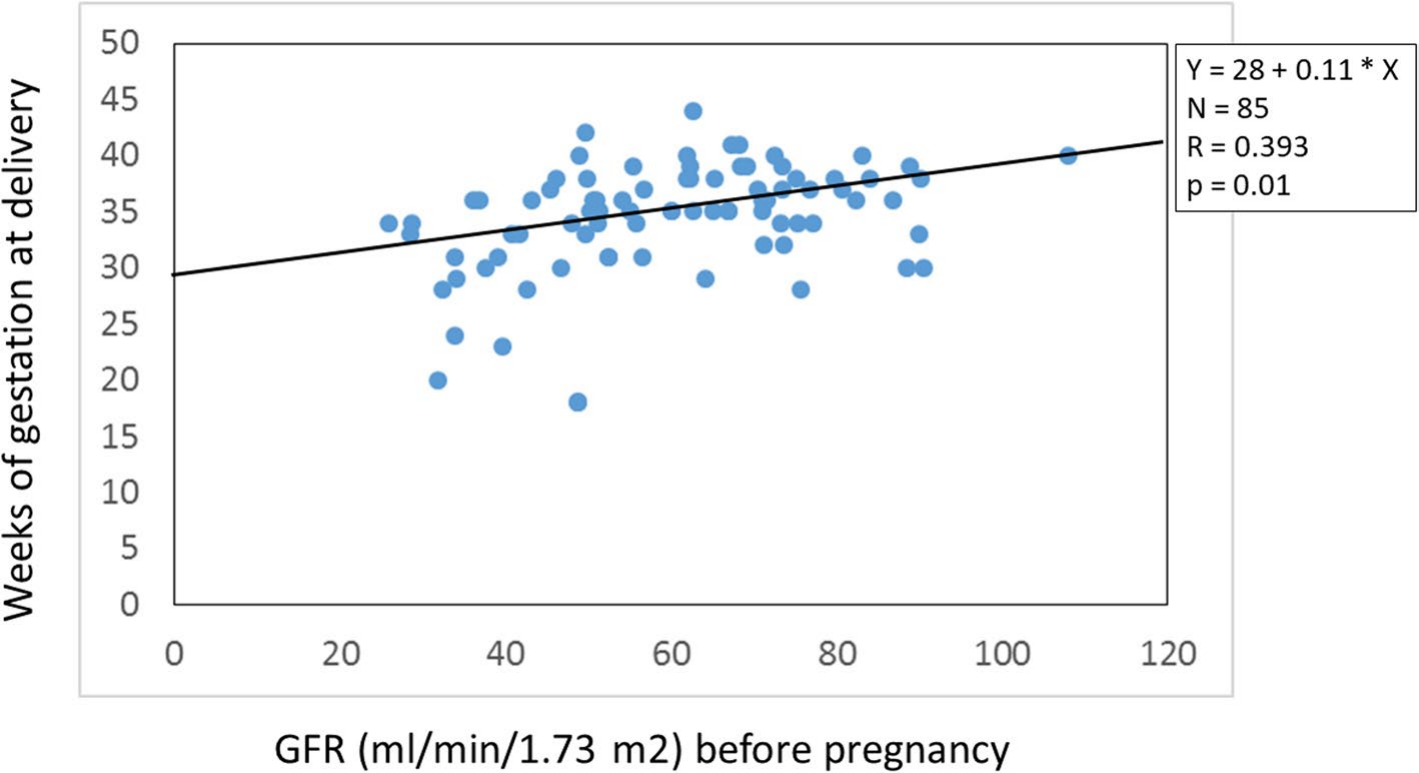
Estimated GFR before pregnancy was correlated with the number of gestation weeks at delivery (*p* = 0.01)

**Fig. 2:**
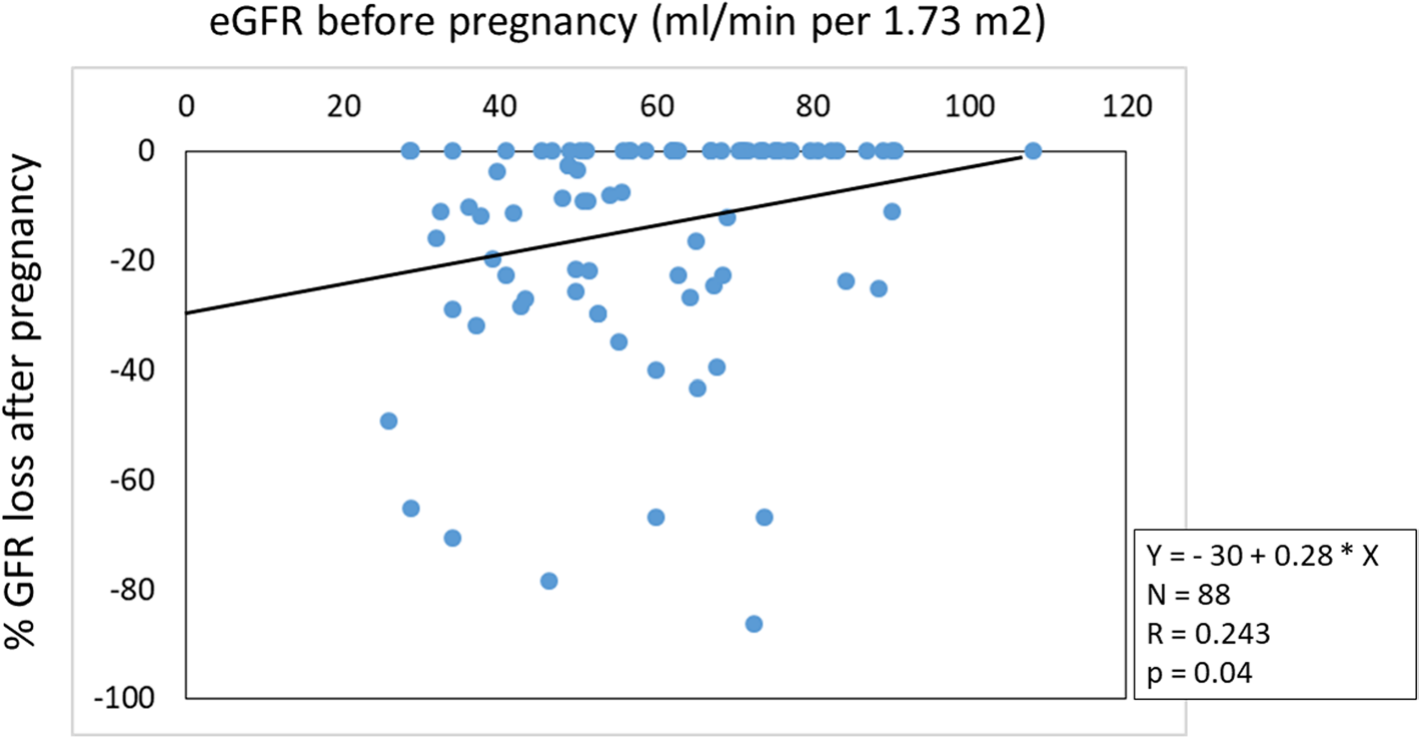
Estimated GFR (eGFR) before pregnancy was correlated with the percent loss of eGFR measured 3–4 months after pregnancy (*p* = 0.04)

Pre-pregnancy eGFR correlated with gestation week at delivery (*R* = 0.393, *p* = 0.01; Fig. 1) and correlated with percent of loss of eGFR measured 3 to 4 months after pregnancy (*R* = 0.243, *p* = 0.04; Fig. 2). Percent of loss of eGFR during pregnancy was inversely correlated with the time from end of pregnancy to chronic graft failure or death measured in months (*R* = -0.47, *p* = 0.001). The age of the transplant (time after transplantation plus donor age at donation) did not correlate to percent of graft loss by pregnancy (*R* = 0.108, *p* = 0.19).

**Fig. 3:**
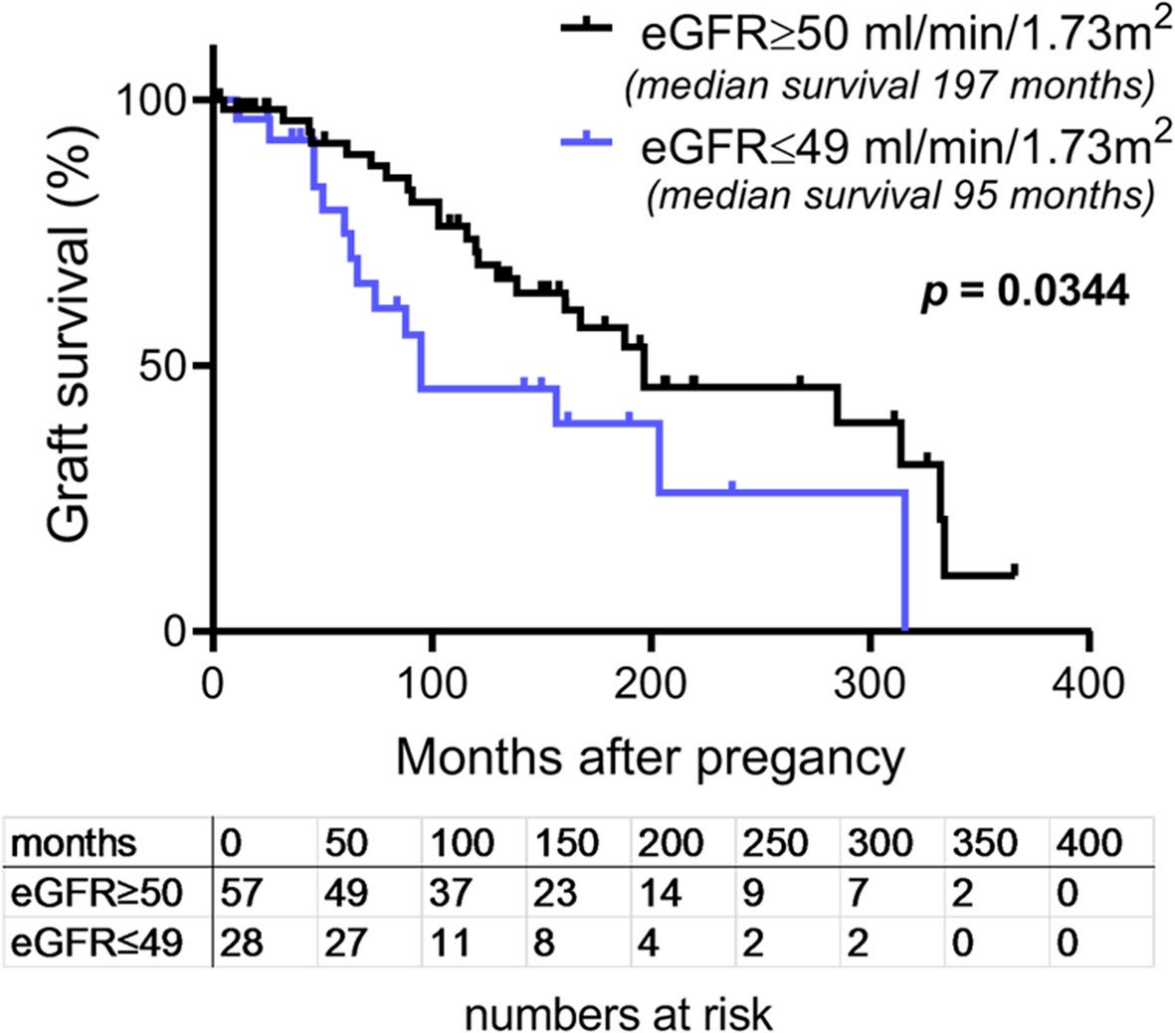
Comparison of graft survival curves of women with pre-pregnancy estimated GFR of ≥ 50 ml/min/1.73 m^2^ to those with estimated GFR of < 50 ml/min/1.73m^2^, Kaplan–Meier curves, Log-rank (Mantel-Cox) test; Chi square 4.475, df 1, *p* = 0.0344

Kaplan–Meier curves comparing women with eGFR before pregnancy of ≥ 50 mL/min/1.73m^2^ with those with eGFR < 50 mL/min/1.73m^2^ showed a significantly longer graft survival after pregnancy in women with an eGFR ≥ 50 mL/min/1.73m^2^ (Log-rank test *p* = 0.0397; Fig. 3). The median graft survival was 197 months with an eGFR > 50 mL/min/1.73m^2^ but only 95 months with an eGFR < 50 mL/min/1.73m^2^. We selected the eGFR cut off value of 50 mL/min/1.73m^2^ for differentiating between a desirable graft function for pregnancy and patients’ actual renal function.

To obtain information about the impact of pregnancy per se on graft function in women with low graft function of > 25 to < 50 mL/min/1.73m^2^, the pregnancy study group and the non-pregnancy control group were compared. Both groups. had a similar basal eGFR (39.6 ± 7.44, *n* = 28, versus 37.77 ± 6.65 mL/min/1.73m^2^, *n* = 79; *p* = 0.228). However, the one-year slope in eGFR 3–4 months after pregnancy expressed as percent of pre-pregnancy eGFR was more pronounced than the one-year slope of eGFR of the non-pregnant group (19.34 ± 22.10%, *n* = 28, versus 2.61 ± 10.95%, *n* = 79; *p* < 0.0001).

Eleven mothers died during 17.75 ± 12.08 years of observation since the first pregnancy in 1972 to end of 2019 (0.75 to 49.17 years). Four deaths were related to infection, 3 to hypertensive or ischemic cardiomyopathy, and 2 were caused by malignant disease (2 others by random causes; Table 3). These women left behind 17 motherless children, of which 16 children were not yet adults at the time of maternal death.

**Table 3:** Age and cause of death of 11 women during observation after pregnancy as well as number and age of children left behind at their mother’s death

**Age of mother at death (yrs)**	**Cause of mothers’ death**	**Age of children left behind at mothers’ death (yrs)**
58	Heart failure by hypertension	24
51	Car accident	16
43	Intraabdominal bleeding of enteric fistula	10, 11
31	Pneumococcal septicemia	5, 8
48	Infected knee prosthesis	12
33	Lymphoma	3, 6
37	Heart failure by hypertension	10
49	Bacterial endocarditis	14
41	Coronary heart disease	12, 13
37	Post-ERCP pancreatitis	1
39	Sarcoma	11, 13, 13

*ERCP* endoscopic retrograde cholangiopancreaticography; patient after liver and kidney transplantation, *yrs* years

## Discussion

The main finding of this study is that pre-pregnancy graft function plays a key role on the outcomes of pregnancy. This state-of-affairs on one hand includes the benefit of the fetus, since pre-pregnancy graft function correlates with the time of gestation until delivery (Fig. 1). On the other hand, it encompasses graft function development, since pre-pregnancy graft function correlates with graft function decline by pregnancy (Fig. 2); and furthermore, the extent of graft function decline engendered by pregnancy correlates with the long-time graft function of the mother after pregnancy up to chronic graft failure or death. This was confirmed by comparing graft survival of the women with pre-pregnancy eGFR ≥ 50 mL/min/1.73m^2^ to < 50 mL/min/1.73m^2^ by Kaplan–Meier curves (Fig. 3). The latter finding may be an argument that renal functional decline engendered by pregnancy mainly reflects the functional reserve of the graft in general. Thus, to tackle the question whether pregnancy after kidney transplantation in women with low eGFR may have an adverse influence on graft function deterioration per se, we compared the change in graft function during the one-year-period of pregnancy with graft function during a one-year-period in a matched non-pregnant control group. The comparison revealed a seven-to-eight-fold higher functional decline during pregnancy, compared to the control group. This result is a strong argument that pregnancy in patients with a low pre-pregnant eGFR has an adverse impact per se on graft function decline. In any event, graft function deterioration is accelerated.

The possible influence of pregnancy on graft function in women after kidney transplantation was the subject of several studies since 1995, mainly 9 single-center studies [8–16]; as well as one multi-center study [17]; one registry study [18]; and one meta-analysis [5]. In these studies, women with one or several pregnancies after renal transplantation were compared to nulliparous transplanted women serving as controls, matched for several important parameters, looking for short- and long-term graft loss and deterioration of renal function. The results of each of the studies as well as the pooled results showed no statistically significant difference between parous and nulli-parous women regarding graft loss and renal function [5]. However, in most of these studies using nulliparous controls the transplanted women with pregnancy and their controls had an excellent renal function at starting point. In three of the studies all patients with pregnancies and nearly all controls underwent living-donor transplantation [10, 11, 13]. The main difference in our study compared to earlier reports is that pre-pregnancy eGFR of the women was rather low: < 50 mL/min/1.73m^2^ in 32% and even < 40 mL/min/1.73m^2^ in 15% of pregnancies.

Armenti, from the National Transplantation Pregnancy Registry, described already in 1995 a concrete threshold of creatinine (1.6 mg/dL or 141 µmol/L, eGFR 45 mL/min/m^2^) above which renal dysfunction during pregnancy has to be expected [19]. This finding was confirmed later by others [20]. However, which level of graft function is adequate to permit a safe and successful pregnancy remains to be established. It seems obvious to transfer the experience with pregnancy associated with chronic kidney disease (CKD) to pregnant patients after renal transplantation in the corresponding stage of renal insufficiency [21]. However, transplanted women with pregnancy have a higher risk, especially in CKD-EPI stage 1 to 3, than CKD patients [22].

An eGFR value > 50 or 60 mL/min/1.73m^2^, or serum creatinine of < 100–120 µmol/L (< 1.1–1.4 mg/dL) before pregnancy is assumed as acceptable for a safe pregnancy, provided that proteinuria is minimal and blood pressure is well controlled [10, 23–28]. The rates of 10–16% electively induced abortions in different countries are influenced by unfavorable conditions of renal graft function, hypertension, and/or severe proteinuria [29]. Nevertheless, other authors report on problems and risks even with a good graft function prior to pregnancy [29–33]. In our experience, the percent of graft functional loss engendered by pregnancy was contingent upon pre-pregnancy eGFR (Fig. 2). This finding implies that an eGFR ≥ 50 mL/min/1.73m^2^ before pregnancy would be relatively safe regarding graft function. However, an eGFR of less than 40 mL/min/1.73m^2^ before pregnancy was not only associated with a high relative mean graft function loss, but was also associated with a high fetal or perinatal death of the offspring. According to our study, the eGFR of 40 mL/min/1.73m^2^ designates the lower limit of a reasonably planned pregnancy.

The cause for the worsening influence of pregnancy on a kidney with low basal renal function is not well studied. It is known that parity with a higher frequency of pregnancy is associated with the risk of albuminuria and chronic kidney disease. This state-of-affairs suggests that pregnancy could be a stress factor for the kidney [34]. Probably hyperfiltration plays a role, which is important for pregnancy outcomes [35]. Possibly a pre-damaged kidney with a restricted number of functioning nephrons that already exhibit hyperfiltration no longer have any available reserves.

Four of 67 women had acute graft loss during 92 pregnancies since 1972. Hemolytic uremic syndrome, which appeared in two cases with acute graft loss, hemolytic anemia, thrombopenia, and acute high blood pressure, may be difficult to differentiate severe preeclampsia with the hemolysis, elevated liver enzymes and low-platelets (HELLP) syndrome even with available biopsy results. In general, we did not find an era effect for the time periods before and after the year 2000 regarding graft and fetal loss as well as graft function decline.

The definition of preeclampsia occurring in 3–5% of normal European pregnancies in general has extended from the traditionally diagnosed combination of hypertension and proteinuria to maternal organ dysfunction, as well as utero-placental dysfunction or fetal growth retardation [36–40]. Differentiation of superimposed preeclampsia from graft-related problems during pregnancy of patients after kidney transplantation is particularly difficult, since the precondition of a renal graft is reduced renal function, often pre-existing hypertension, and a certain amount of pre-existing proteinuria [39–41]. The diagnostic accuracy can be improved by incorporating novel biomarkers, such as soluble *fms*-like tyrosine kinase 1 (sFlt1) to placental growth factor (PIGF) ratio. This method may identify preeclampsia 5 weeks prior to the onset of symptoms [39, 42–44]. In our experience, preeclampsia was the leading cause of fetal or perinatal death, which has been reported by others [45]; preeclampsia also was the cause of acute graft failure in one case. Besides the high rate of preeclampsia, other well-known complications related to the renal transplant status of the mother were the shortness of gestation with premature delivery, low birthweight infants, and the high rate of cesarean section. [25, 29, 31, 33].

Aside from one woman who lost her graft because of noncompliance, we did not observe severe rejection episodes during or shortly after pregnancy. A low rejection rate has been reported by other authors as well [29, 31]. However, as pregnancy is known to have an alloimmunization risk by initiating HLA antibodies we would have expected a higher rejection rate [46, 47].

We were surprised that the age of the transplant, including time from grafting plus donor age at donation, that was up to 67 years in our patients, did not correlate with the functional decline caused by pregnancy. This finding suggests that a transplanted kidney of an “older” donor can survive a pregnancy several years after transplantation, if pre-pregnancy renal function is good. The usually recommended timepoint of pregnancy is one to two years after transplantation; this is not realistic considering that 21% of our here presented women were transplanted during their childhood [48–52]. These women desired to have a child when they attained adulthood. In our cohort, the mean transplant to conception interval was 6.51 ± 5.33 (0.5–24.75) years, which is in the higher range of reported transplant to conception intervals [5].

An interesting side aspect of our study is the fact that since 2000 nearly 40% of the women with a serious desire to become pregnant (they had altered their medication after counseling) did not succeed in their wish to sustain a pregnancy beyond the third trimester. The cause of not becoming pregnant was not analyzed in these women. However, the observed 40% infertility corresponds to the post-transplant fertility rate given in the literature of around 60–70%. As a matter of fact, fertility rate of women after kidney transplantation is two-thirds of childbearing-aged women in the general population [29, 53].

Most of the transplanted women completing a successful pregnancy were young and healthy. However, 16% of our patients after pregnancy died 1 to 24 years thereafter. Most succumbed to the typical causes of death occurring to patients with end-stage renal disease, infections, ischemic or hypertensive cardiomyopathy, and malignancy. Most of the children were not yet adults at the time of maternal death.

## Conclusions

In counseling transplanted women concerning pregnancy an open and frank discussion is necessary [54]. It is known that pre-pregnancy renal function should be “good”, however, the lower limit usually is not given. We believe that our findings provide some guidelines in terms of how we should counsel women.

First, we should tell the women that their fertility rate is not normal but reduced by approximately one third. Second, that good renal function is a precondition for the benefit of the fetus by prolonging the period of gestation and allows bringing the pregnancy to term. Third, we are obligated to inform the women that most cases of fetal death were observed in women with a pre-pregnancy graft function of less than 40 mL/min/1.73m^2^. The women also should be aware that pre-pregnancy graft function correlates with a relative loss of graft function after pregnancy, as well as the time to graft failure in general after pregnancy and delivery. The decrease in graft function in patients with a low pre-pregnant graft function may be accelerated by pregnancy. According to our findings, the time interval from transplantation to pregnancy and the “age” of the graft including donor age are less important than pre-pregnancy graft function. In our study, the time interval was up to 20 years in women transplanted as children. The counseling should include as far as possible that the planned pregnancy and childhood needs a stable family situation.

## Data Availability

Data were obtained from the medical records. The datasets generated and analysed during the current study are not publicly available since individual privacy could be compromised. They are available from the corresponding author on reasonable request.

## Abbreviations

CAKUT: Congenital anomaly of the kidney and the urinary tract
CKD-EPI: Chronic Kidney Disease Epidemiology Collaboration
CTG: Cardiotocography
eGFR: Estimated glomerular filtration rate
vs: Versus
HELLP: Hemolysis, high liver enzymes, low platelet count
sFlt-1: Soluble fms-like tyrosine kinase 1
PIGF: Platelet derived growth factor
